# Is There a Common Summary Statistical Process for Representing the Mean and Variance? A Study Using Illustrations of Familiar Items

**DOI:** 10.1177/2041669517747297

**Published:** 2018-01-25

**Authors:** Yi Yang, Midori Tokita, Akira Ishiguchi

**Affiliations:** Graduate School of Humanities and Sciences, Ochanomizu University, Tokyo, Japan; Faculty of Health Sciences, Mejiro University, Saitama, Japan; Faculty of Core Research, Ochanomizu University, Tokyo, Japan

**Keywords:** summary statistical representation, individual difference, mean, variance

## Abstract

A number of studies revealed that our visual system can extract different types of summary statistics, such as the mean and variance, from sets of items. Although the extraction of such summary statistics has been studied well in isolation, the relationship between these statistics remains unclear. In this study, we explored this issue using an individual differences approach. Observers viewed illustrations of strawberries and lollypops varying in size or orientation and performed four tasks in a within-subject design, namely mean and variance discrimination tasks with size and orientation domains. We found that the performances in the mean and variance discrimination tasks were not correlated with each other and demonstrated that extractions of the mean and variance are mediated by different representation mechanisms. In addition, we tested the relationship between performances in size and orientation domains for each summary statistic (i.e. mean and variance) and examined whether each summary statistic has distinct processes across perceptual domains. The results illustrated that statistical summary representations of size and orientation may share a common mechanism for representing the mean and possibly for representing variance. Introspections for each observer performing the tasks were also examined and discussed.

## Introduction

Variation is a major feature of the natural world. For example, grapes in a cluster appear similar, but upon closer examination, differences in colour, size and shape are apparent; similarly, children in the same age-group may resemble each other, but they exhibit large differences in size, character and mental states. Under these circumstances, people can categorise objects, events and living states in our environment, estimate the average value or extent of variation and prepare for adaptive decision-making. Many studies have described this visual ability as a *statistical summary representation* or ensemble representation, and researchers in several fields have explored behavioural characteristics (e.g. Alvarez, 2011; Ariely, 2001; Barlow & Tripathy, 1997; Brady, Shafer-Skelton, & Alvarez, 2017; Chong & Treisman, 2003; Haberman, Brady, & Alvarez, 2015; Morgan, Mareschal, Chubb, & Solomon, 2012) as well as the neurophysiological basis for such representation systems (Cant & Xu, 2012; Leib et al., 2012).

It is important to note that studies of summary statistical representation can be roughly categorised into two approaches: the psychophysical approach (e.g. Barlow & Tripathy, 1997; Dakin & Watt, 1997; Watamaniuk, 1993) and the cognitive approach (e.g. Alvarez, 2011; Ariely, 2001; Brady et al., 2017; Chong & Treisman, 2003; Haberman et al., 2015). These two approaches differ in the property of stimuli, the number of stimuli in a set, and the experience of an observer of the mean extraction task.

The psychophysical approach has mainly used textures or random dots as stimuli; stimuli sets are composed of considerably large number of simple items including dots and Gabor patches; a few experienced observers participated in the experiment with a large number of trials; the efficiency with which observers could extract summary statistics is tested using the ideal observer analysis. Some psychophysical studies share stimuli with cognitive approach (Solomon, Morgan, & Chubb, 2011; Tokita, Ueda, & Ishiguchi, 2016). For example, Solomon et al. used eight circular discs as stimuli set.

However, the cognitive approach, with which the present study concerns, has used items more complicated than the dots or Gabor patches, including circles with different sizes, different facial expressions, sequence of different tones and others; the number of stimuli in a set ranged from 2 to 16, which is considerably smaller than those in the psychophysical approach; a large number of inexperienced observers participated in the experiment and the efficiency of extraction of summary statistics is tested using the precision of the extraction of mean values across the set size and presentation time. Noticeably, it has been suggested that there are three pieces of behavioural evidence for the efficient extractions of summary statistics. First, the precision of extraction of mean values remains constant regardless of the number of stimuli in a set (Chong & Treisman, 2003, 2005; Haberman & Whitney, 2009), suggesting that the mean value is not calculated one at a time. Second, the precision of mean values is not affected by the presentation time (Chong & Treisman, 2003; Leib, Kosovicheva, & Whitney, 2016). Besides, Chong & Treisman (2003) demonstrated that observers could extract the mean value of the stimuli in a set at a presentation time as low as 50 ms. Third, the mean value is extracted even when information of an individual item is not recognised by the observer, suggesting that summary statistics of stimuli in a set may be computed without representing individual items (Ariely, 2001; Chong & Treisman, 2003; Tokita et al., 2016).

Previous researches in the cognitive approach illustrated that observers can extract statistical values over a range of visual properties, including size (Alvarez, 2011; Ariely, 2001; Chong & Treisman, 2003; Oriet & Brand, 2013), position (Alvarez & Oliva, 2008) and emotional expression (Haberman & Whitney, 2009, 2011). This ability is not limited to static and simultaneous events, as it is observed in sequentially presented events (Albrecht, Scholl, & Chun, 2012; Corbett & Oriet, 2011; Hubert-Wallander & Boynton, 2015) and dynamic objects, such as expanding and contracting circles (Albrecht & Scholl, 2010). Moreover, the ability to represent statistical properties is not only limited to visual properties but also observed for auditory properties such as extracting frequency information from sequences of sounds (Piazza, Sweeny, Wessel, Silver, & Whitney, 2013) and temporal variation of sounds (McDermatt, Schemisch, & Simoncelli, 2013). These representations of statistical properties have been suggested to assist our judgement and behaviour more efficiently than assessing each object or event individually (e.g. Alvarez, 2011; Ariely, 2001, 2008; Chong & Treisman, 2003; Robitaille & Harris, 2011). This ability may preserve cognitive resources, such as memory and selective attention load, and provide rich information about the environment rapidly and parsimoniously.

Although there is an understanding of the possible existence of general summary representation systems (e.g. Alvarez, 2011; Ariely, 2001, 2008; Chong & Treisman, 2003), it has not been tested whether different types of summary statistics share a common system or whether dedicated systems exist for different summary statistics. Summary statistics include the mean, variance, skewness, kurtosis and correlation (when more than one variable is observed). Nevertheless, most studies investigated the mean, and some studies examine the variance of items in a set; however, the relationship between these statistics has not been tested. This, therefore, poses the question of whether different types of summary statistics share a common system or have distinct processes. For instance, is there a common mechanism supporting all summary statistic types, or are multiple mechanisms for a specific summary statistic at work? When we calculate the variance of a data set formally, we must first determine the mean value of the set. Does the statistical summary system work in the same manner or, unlike formal calculation, do distinct processes exist?

To clarify whether the summary statistical process is supported by a common or separate mechanism(s), we employed an individual difference approach. The approach has been employed by many studies that explored the functional organisation of cognitive mechanisms (Haberman et al., 2015; Huang, Mo, & Li, 2012; Underwood, 1975; Wilmer, 2008). Individual difference approaches are particularly useful for addressing questions of cognitive processes because they take advantage of the intrinsic variability present in a population sample (Bosten et al., 2015; Bosten & Mollon, 2010; Huang et al., 2012). By examining how performance on different tasks is correlated, we can infer whether such processes are likely supported by a common underlying mechanism or independent operating mechanisms. For example, Haberman et al. (2015) used an individual difference approach to define the functional organisation of ensemble perception, testing whether the cognitive processes between high- and low-level ensemble representations are correlated.

In line with the approach, if the results from the mean and variance representation tasks are strongly correlated, then it is predicted that the mean and variance are supported by a common mechanism. However, the lack of a correlation indicates that different processes are involved in representing the mean and variance. Specifically, if different summary statistics share a common system, then an individual who can precisely represent the mean value of a set of items should also be able to precisely represent the variance of the set. That is, there should be a relationship regarding performance in mean and variance representation tasks. If a positive relationship is observed, then it is predicted that a common summary statistics mechanisms exists for representing the mean and variance of a set of items. Alternative, distinct mechanisms for the mean and variance suggest that multiple summary statistical processors specific to each type of statistical value exist. In other words, there are separate ‘cognitive mechanisms’ for representing the mean and variance.

Concerning the introspections of observers, we asked them to describe how they performed each task at the end of the experimental session. Although the explicit statements may not necessarily predict the exact behavioural processes, it would be useful to know the strategy observers employed at the conscious level.

Concerning the perceptual domains of items, we used size and orientation in this study, as both domains have been well examined in mean and variance tasks (Chong & Treisman, 2003; Morgan et al., 2012; Parkes, Lund, Angelucci, Solomon, & Morgan, 2001; Solomon et al., 2011; Tanaka & Ishiguchi, 2006; Tokita et al., 2016). In the size and orientation domains, most studies used discs and Gabor patches, respectively; however, we introduced more familiar and realistic items such as illustrations of strawberries as the size stimuli and those of lollypops as the orientation stimuli. The reason was to test whether an ability of the *statistical summary representation* extends to a wide range of objects, as objects in the natural world are multidimensional. It is important to know whether observers could extract summary statistical values of illustrations with the same precision as observed for more primitive objects. Thus, in addition to the primary objective, we explored whether representing the mean and variance of sets of complicated items (i.e. illustrations of strawberries and lollypops) could be represented in equivalent precision to simple items such as discs and Gabor patches. When we compared the results of complicated items with the simple items, we used the results of the simple items in previous research because we did not use the simple items in this study.

Along with exploring the relationship between the mean and variance in size and orientation domains, we tested the relationship between performances in size and orientation domains for each summary statistic (i.e. mean and variance) and examined whether each summary statistic has distinct processes across perceptual domains. Regarding this issue, Haberman et al. (2015) revealed no correlation between the mean representation of high- (i.e. emotional expression) and low-level items (i.e. orientation), thus arguing against a single domain-general ensemble mechanism. We tested this possibility using size and orientation domains. If performances in the size and orientation domains in the mean or variance tasks are correlated, then the mean or variance may be represented via a common process.

Thus, this research had one primary and two secondary objectives, and it was conducted using an individual differences approach. The primary objective was to test whether different types of summary statistics (i.e. mean and variance) share a common system or whether dedicated systems exist for each statistical value. In addition, we explored whether representing the mean and variance of sets of complex items could be represented with equivalent precision to that for simple items such as discs and Gabor patches. Moreover, we tested whether a single domain-general mechanism supports multiple statistical representations or whether domain-specific mechanisms are at work for the mean and variance.

## Experiment

Observers performed four tasks, namely mean and variance representation tasks combined with size and orientation domain conditions. All tasks were performed with a within-subject design. Four measures were gathered. First, the correct rate for each comparison level was calculated, and the discriminability threshold of each task in each perceptual domain was obtained. Second, the correlations between performances in the mean and variance representation tasks in each domain were calculated. Third, the correlation between performances in the size and orientation mean tasks and that between performances in the size and orientation variance tasks were calculated. In addition, introspections were obtained from each observer at the end of experimental sessions.

## Method

### Participants

A total of 32 observers (19 females and 13 males), all with normal or corrected-to-normal visual acuity, participated in all tasks. Fourteen observers were undergraduate students from Mejiro University, the others were undergraduate and postgraduate students from Ochanomizu University.

All observers provided informed consent prior to participation, and they were not informed of the purpose of this study. This research was approved by the Mejiro University’s institutional review board.

### Apparatus

The experiment was conducted in a normally lit room. Stimuli were displayed on an iMac desktop computer monitor controlled by a Macintosh computer (Mac OS X). Stimuli were generated using Psychophysics Toolbox Version 3 (Brainard, 1997; Kleiner et al., 2007; Pelli, 1997) for MATLAB (Version 8.4, Mathworks, MA). Observers viewed the screen with both eyes, and they were seated approximately 60 cm from the screen.

### Design

We considered two perceptual domains (size and orientation) and two types of summary statistics (mean and variance). Concerning each domain, the participants were required to perform the mean representation task (i.e. mean task) and variance representation task (i.e. variance task). Thus, there were four tasks in total. Each participant performed all four tasks, yielding within-subjects designs across two perceptual domains for two summary statistics.

Each task comprised standard and comparison stimuli. We introduced four comparison levels in each task. At the end of experimental sessions, each participant answered questionnaires about how he or she performed the tasks.

### Stimuli

**Figure 1.**
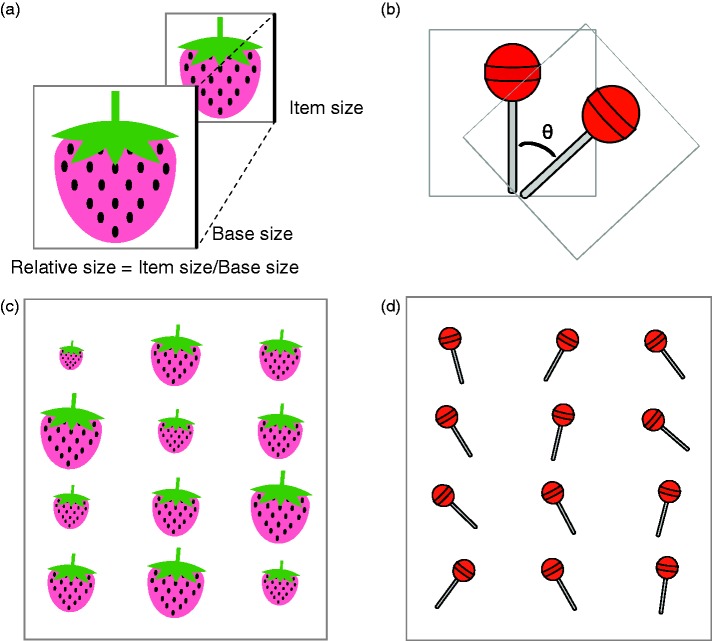
Two types of items: a strawberry for the size domain and a lollypop for the orientation domain. (a) and (b) Illustration of how size and orientation were controlled for each domain. (c) and (d) Examples of stimulus sets.

Figure 1 shows examples of item sets. Regarding the item for the size domain, we used an illustration of a strawberry created using PowerPoint. The item for the orientation domain was an illustration of a lollypop, also created using PowerPoint. All items were presented against a light grey background.

All parameters used in both tasks were decided on the basis of pilot studies. To control the variance of item sets, we used a fixed variance generation method in which the mean and SD of the samples randomly drawn from normal distribution were fixed to the expected value (Tokita & Ishiguchi, 2015).

The items of each domain were placed in a square cell with specific side lengths. The array was divided into a 4 × 3 matrix. Each cell subtended a visual angle of 1.16° × 1.16°. The entire array subtended 5.56° × 6.25°. The items were arranged within the array. Each item was displayed at the centre of each cell.

#### Size domain

Figure 1(a) and (c) presents examples of items for the size domain. The size of an item was defined by a square of the side length of the item. Henceforth, side ‘length’ denotes ‘size’ in this domain for simplicity. The mean size of items in a set was represented as 1, and the size of each item in a set was expressed relative to the mean size. Variance was expressed as previously described (Tokita et al., 2016).

**Figure 2.**
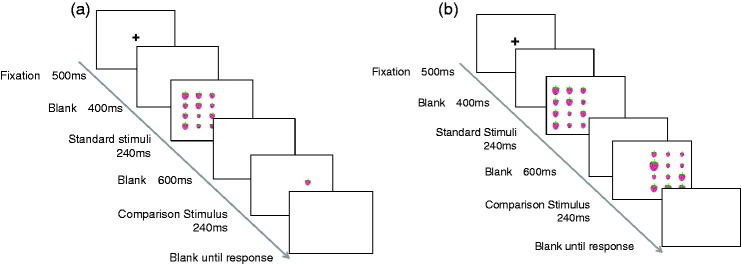
Schematic diagrams of trial sequences in the mean (a) and variance tasks (b) for the size domain.

In the mean task, two stimuli were presented sequentially, namely standard and comparison stimuli, in each trial (Figure 2(a)). The standard set consisted of 12 items (i.e. an illustration of strawberries) and the comparison stimulus consisted of a single item. In the standard stimuli, a set of 12 items was presented in the array area. Lognormal Gaussian noise *lnN* (*lnLength*, σ_size_^2^), in which length and σ_size_ represent the base length and *SD* of the distribution, respectively, was added to the length of each item of a set independently. It has been established that a lognormal distribution of circle diameters will produce a Gaussian distribution of discriminable sizes after logarithmic transduction (Solomon et al., 2011). Although the items in this study were not discs, we introduced the same methods because both tasks dealt with area.

The size variance of the standard stimuli was fixed to a square of 0.14 in the relative size scale. There were four comparison levels, namely squares of 0.87, 0.93, 1.07 and 1.05, relative to the mean size of the standard set.

In each trial, all of the presented items were randomly scaled by a small multiplicative factor to discourage the observers from basing their judgements on previously viewed items. Four multiplicative factors (1, 1.05, 1.1 and 1.15) were used, and the same factor scaled all items in any one trial.

Regarding the variance task, schematic views of stimulus presentation are shown in Figure 2(b). Stimulus presentation followed that described for the mean task except that both arrays (i.e. standard and comparison sets) comprised 12 items. The array positions of the standard and comparison sets were changed randomly; in half of the trials, the standard set appeared on the left side while the comparison set appeared on the right side, or vice versa.

The size variance of the standard set was fixed to a square of 0.14, as described for the mean task. The size variances of the comparison sets were squares of 0.17, 0.20, 0.23 and 0.26; thus, there were four comparison levels.

In each trial, the sizes of the item in a set were randomly scaled by a same multiplicative factor as described for the mean task. The added scale factor could differ between the standard and comparison sets; thus, the absolute mean size of items in a comparison set could differ from that in the standard set. Specifically, the variance was controlled on the basis of the relative sizes of items. This operation was necessary to ensure that observers would perform the task by truly extracting the size variance of items and not by referring to the size of outliers.

#### Orientation domain

**Figure 3.**
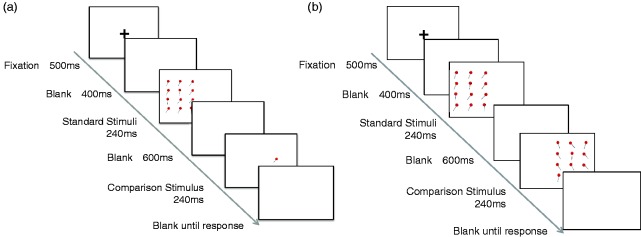
Schematic diagrams of trial sequences in the mean (a) and variance tasks (b) for the orientation domain.

Stimulus presentation followed that for the size domain. The orientation of the sticks of lollypops varied (Figure 1(b)). Schematic diagrams of the trial sequence in each task are shown in Figure 3(a) and (b), respectively.

In the mean task, the base orientation of the items in the standard set was randomly chosen from orientations ranging from −30° to +30° relative to vertical (−: counterclockwise; +: clockwise). Gaussian noise *N*(0, σ_orien_^2^) was added to the orientation of each item of a set independently. The orientation variance of the standard stimuli was fixed to a square of 13°. There were four comparison levels: −14°, −8°, 8° and 14° relative to the mean orientation.

In the variance task, stimulus presentation followed that in the mean task except that both arrays (i.e. standard and comparison sets) comprised item sets. The original orientation of the items in the standard and comparison sets was randomly and independently chosen from orientations ranging from −30° to 30° relative to vertical. Thus, the original orientation could differ between the standard and comparison sets. Gaussian noise *N*(0, σ_orien_^2^) was added to the orientation of each item of a set independently. The variance of the standard stimuli was a square of 13°. The variances of the comparison stimuli were squares of 16°, 19°, 22° and 25°; thus, there were four comparison levels. Note that although the proper way of generating the orientation variance may be with von Miles circular statistics, we used a conventional way of variance computation, as the same way in the orientation variance discrimination task in previous studies (Morgan, Chubb, & Solomon, 2008; Solomon et al., 2011). To test the validity of using the normal distribution in calculating the variance, we compared the probability density function of von Mises circular statistics to that of normal distribution with the variance values used in the present study and demonstrated that the von Mises distribution with this variance value approximate to the normal distribution.

### Procedure

A schematic view of stimulus presentation is shown in Figures 2 and 3. Observers completed one 50-min session that consisted of a practice block of eight trials, followed by four experimental blocks of 80 trials each (4 comparison levels × 20 repetitions), thus resulting in a total of 320 trials. The comparison levels and the order of trials were randomly mixed. The order of blocks was counterbalanced across observers using a balanced Latin square, which controls for order effects. Observers were required to obtain correct rate of 100% in the practice trials to ensure they fully understood the task instructions. A two-alternative forced choice procedure was used in all tasks.

Each trial started with the display of a fixation cross for 500 ms followed by a blank screen for 400 ms. The items in a set were first presented for 240 ms. The comparison item was presented for 240 ms after a blank screen for 600 ms, and then a blank screen was shown until a response was recorded. The next trial automatically began 500 ms after the response. In the mean task for the size domain, observers were asked to decide whether the comparison item was larger or smaller than the mean size of items in a standard set. When they thought that the comparison item was smaller than the mean size of the item set, they pressed the right arrow key; otherwise, they pressed the left arrow key. In the mean task for the orientation domain, observers were asked to decide whether the orientation of the comparison item (i.e. sticks of lollypops) was clockwise or counterclockwise to the mean orientation of the standard set. When they thought that the comparison item was clockwise to the mean orientation, they pressed the right arrow key; otherwise, they pressed the left arrow key.

In the variance task, observers were asked to decide which item set in a sequence had a larger variance in size or orientation. When they thought that the variance on the right array was larger than that on the left array, they pressed the ‘z’ key; or otherwise, they pressed the ‘c’ key.

No feedback about the correctness of the response was provided for any of the trials. The correct rate on each block was presented at the end of the block to motivate each observer to fully engage in the experiment.

### Analysis

We calculated the mean correct rates. We used these measures and performed the following four analyses. First, we examined how precisely the observers represented the mean and variance. In the size condition, the Weber fraction (Wf) and point of subjective equality (PSE) were measured. In the orientation condition, just noticeable difference (JND) and PSE were measured using the method of constant stimuli. The sizes or orientations for the comparison item were plotted on the *x-axis*, and the proportion of larger or clockwise responses for each comparison stimulus was plotted on the *y-axis*. Fits of psychometric function were done for means across observers. The plotted data points constructed the psychometric function approximated using a cumulative Gaussian function. Wf and JND were defined as the smallest stimulus number change for which a correct response rate of 75% was achieved. The PSEs were obtained as the values of the locations on the psychometric function at which the standard and comparative choice probabilities were equal to 50%. Second, the correlations between the total correct rates in the mean and variance tasks for each domain were tested. Third, the correlations between the total correct rates in the size and orientation domains for each summary statistic were tested.

## Results

Data for two observers were excluded as follows. One observer did not understand the instructions, as his or her correct rate under the difficult condition was higher than those under easier conditions, which were less than 50%, suggesting that the observer had responded oppositely. The other observer appeared to have difficulty in performing the mean orientation task, as she or he needed more than 3 s to respond in 8 of 20 trials. Thus, data were collected for 30 observers.

### Performance for the Mean and the Variance Tasks

#### Size domain

**Figure 4.**
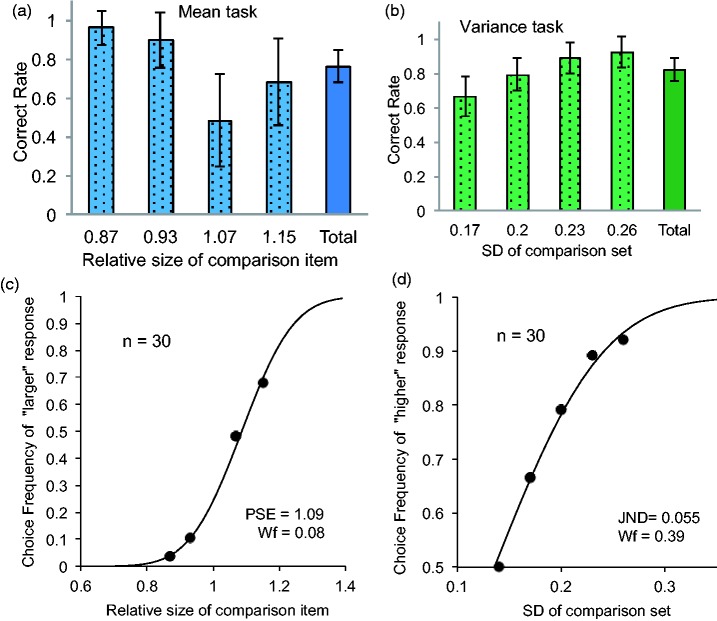
Correct rate at each comparison level and the mean rate (total) in the mean and variance tasks in the size domain (a, b). Psychometric functions in the mean and variance tasks (c, d).

Figure 4(a) shows the correct rate for each comparison level and the mean rate for the mean task. Figure 4(c) shows the psychometric function created by the mean of choice frequency of ‘larger’ responses at the comparison level. The Wf of the mean task was estimated to be 0.08 (an 8% side length difference), whereas the PSE was estimated to be 1.09. The Wf value was almost equivalent to the precision of averaging tasks in several studies that used simple circular stimuli and found a diameter distance of 6% to 8% for a 75% correct rate (Ariely, 2001; Chong & Treisman, 2003; Tokita et al., 2016). Thus, it is indicated that observers could extract a mean value from a set of complicated objects such as strawberries with the same accuracy observed for simpler circular items. The value of PSE was larger than 1, suggesting that the average size of items in a set was overestimated compared with the comparison size. The result was consistent with that of Tokita et al. (2016), who found that a naïve observer tended to overestimate the mean size of a set of circular items.

Figure 4(b) shows the correct rate for each comparison level and the mean rate for the variance task. Figure 4(d) shows the psychometric functions created by the mean ‘larger’ response at each comparison level. The Wf was estimated to be 0.39 (in *SD*). The Wf value was almost equivalent to the precision of the variance discrimination task in previous studies that used discs as items in a set (Tokita et al., 2016; Ueda, Tokita, & Ishiguchi, 2014). In this study, the Wf was ranged from 0.35 to 0.42 for a 75% correct rate. Thus, it is indicated that observers could discriminate the variance between sets of items such as strawberries, with the same accuracy observed for simpler circular items.

#### Orientation domain

**Figure 5.**
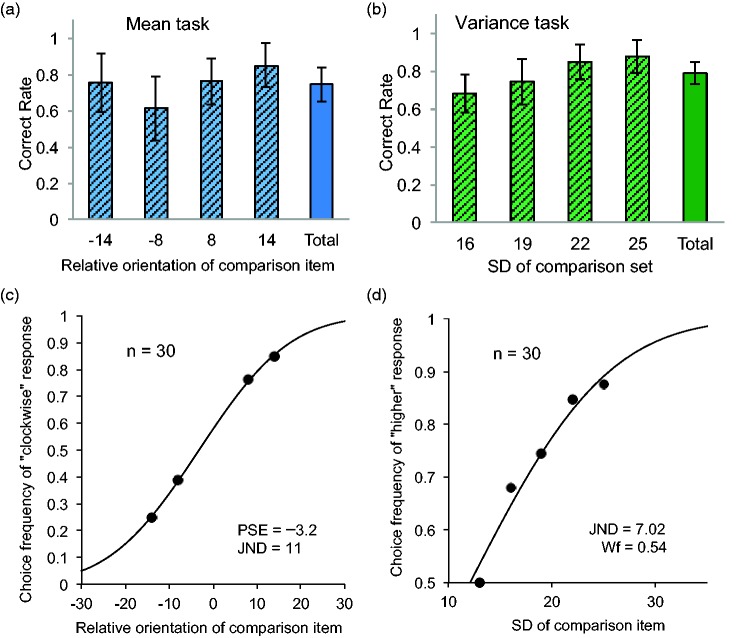
Correct rate at each comparison level and the mean rate (total) in the mean and variance task in the orientation domain (a, b). Psychometric functions in the mean and variance tasks (c, d).

Figure 5(a) shows the mean correct rates of observers along each comparison level and the mean rate. Figure 5(c) shows the psychometric functions created by the mean correct rate at each comparison level. The JND of the mean task was estimated to be 10.9°, whereas the PSE was estimated to be −3.20°. The JND was almost equivalent to the precision of averaging tasks in a previous study that used Gabor patches (Yang, Tokita, & Ishiguchi, 2016). In their study, the JND was ranged from 8.6 to 11.5. Thus, it is indicated that observers could extract the mean orientation of a set of items such as lollypops with the same precision observed for Gabor patches. The PSE was smaller than 0, suggesting that the average orientation of a set of objects was slightly biased toward the clockwise direction. As bias was not systematically tested in any previous research, further investigation is necessary to obtain the general picture.

Figure 5(b) shows the correct rate at each comparison level and the mean response rate for the variance task. Figure 5(d) shows the psychometric functions created by the average correct rate at each comparison level. The JND of the variance task was estimated to be 7.02°. The JND was also similar to the precision of the variance discrimination tasks in previous studies that used simple Gabor patches (Tokita & Ishiguchi, 2015; Ueda et al., 2014). Thus, it is indicated that observers could discriminate the variance between sets of objects such as lollypops with similar precision as observed for simpler stimuli such as Gabor patches.

### Correlation Between the Mean and Variance Tasks in Each Domain

**Figure 6.**
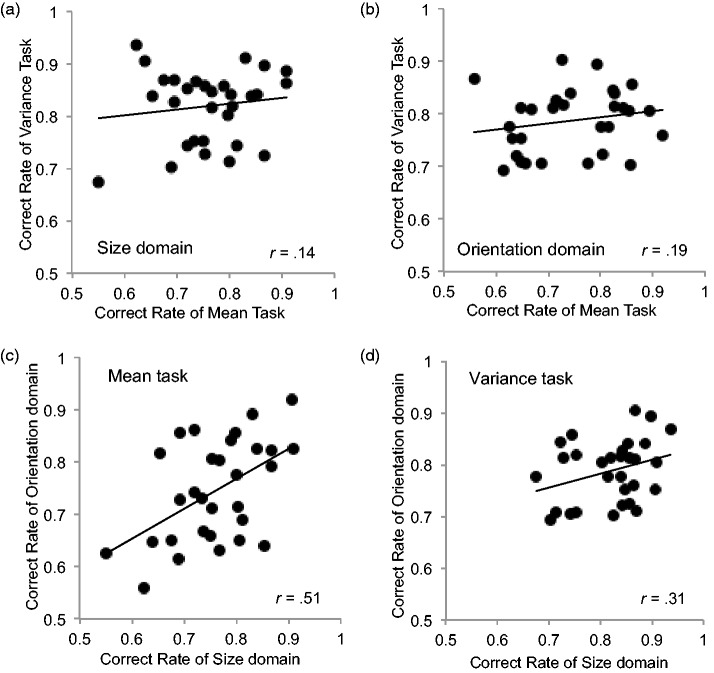
The correlation between the mean and variance tasks in the size and orientation domains (a, b). The correlation between the size and orientations domains across tasks (c, d).

Figure 6(a) and (b) shows the correlation between performances in the mean and variance tasks in the size and orientation domains, respectively. As shown in the figure, correlations between the mean and variance tasks were extremely weak in the size (*r* = .14, *p* = .37, 95% confidence interval [CI] = −0.24–0.47) and orientation domains (*r* = .19, *p* = .32, 95% CI = −0.18–0.51). Thus, precision in extracting the mean size of items in a set does not predict an observer’s ability to represent size variance. This was also the case in the orientation domain. The results imply that representations of mean and variance may involve independent, distinct processes across perceptual domains.

### Correlation Between the Size and Orientation Domains for Each Summary Statistic

Figure 6(c) and (d) shows the correlations between performances for the mean task in the size and orientation domains and those for the variance task in the size and orientation domains, respectively. As shown in Figure 6(c), there was a moderate and significant correlation for individual performances between the size and orientation domains in the mean task (*r* = .51, *p* < .01, 95% CI = 0.18–0.73). Thus, precision in extracting the mean orientation of items in a set moderately predicts an observer’s ability to extract the mean size of an item set. The results suggest that representation of the mean may be a general process, at least for the size and orientation domains.

As shown in Figure 6(d), although nonsignificant, there was also a moderate correlation between the size and orientation conditions in the variance task (*r* = .31, *p* = .09, 95% CI = −0.06–0.60). Thus, it is uncertain whether precision in extracting the orientation variance of items in a set may predict an observer’s ability to precisely extract the size variance.

### Classification of Comments for Each Task

**Figure 7.**
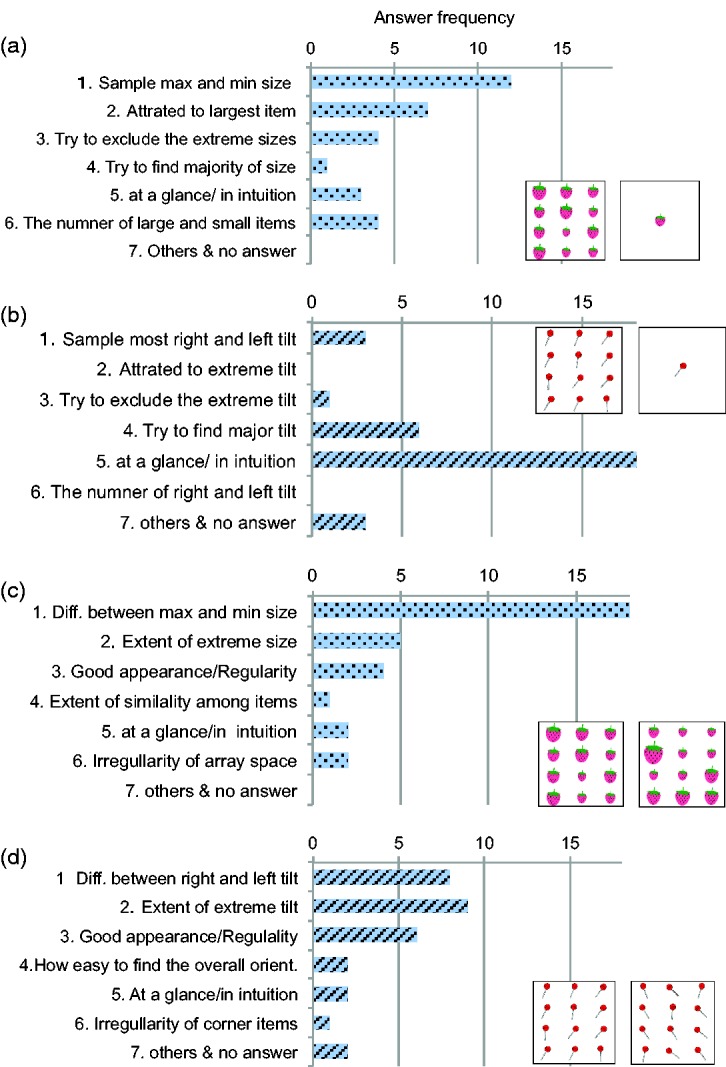
Classification of the questions in each task: answers for the mean (a, b) and variance tasks (c, d) for the size and orientation domains.

All observers were asked to complete a form consisting of four questions: (a) How did you estimate the mean size of a strawberry? (b) How did you discriminate the variance of strawberry sizes? (c) How did you estimate the mean orientation of lollypops? (d) How did you discriminate the orientation variance? The classifications of answers in each task are shown in Figure 7. We classified all answers using the keyword extraction method, which has been used to classify free descriptive data obtained from questionnaires (Beliga, 2014; Kimura, 1995). Briefly, we created a database of answers for each questionnaire and summarised the answers, and we extracted keywords based on usage frequency and relevance to the tasks. Then, we organised the keywords to classify each answer to the group with similar keyword usage. Overall, the observers appeared to use various strategies to perform each task. For both tasks in the size domain, observers commented that they focused on the maximum and minimum sizes of strawberries and used the information to perform the tasks. Some observers noted that they focused on the larger items and found it difficult to ignore them. Conversely, in the mean orientation task, most observers commented that they make decision ‘at a glance’ or ‘on intuition’, suggesting that they did not have particular strategy for extracting the mean. In the orientation variance task, observers stated that they focused items with extreme orientations, which was similar to the strategy used for the variance task in the size domain.

## Discussion

We explored whether different types of summary statistics shared a common representation system or whether a dedicated system was employed for each statistic. In this experiment, we focused on the mean and variance of items in a set as summary statistics in comparison and introduced size and orientation as the perceptual domains of the items. To address the issue, we employed an individual-differences approach that enabled us to infer whether a certain process is supported by a common underlying mechanism or involved distinct mechanisms (e.g. Haberman et al., 2015; Huang et al., 2012). We predicted that if different summary statistics share a common system, then an individual who can precisely represent the mean values of the set should also be able to precisely represent the variance of the set. Namely, there would be considerable correlation between performances in the mean and variance tasks. Our results revealed no significant correlation between performances in the mean and variance tasks for both domains, implying that the derivation of the mean and variance are mediated by the distinct representation mechanisms for human observers.

Thus, we could conclude that estimation of the mean and variance may involve distinct underlying mechanisms, at least for size and orientation domains. This does not deny the possibility of common underlying mechanisms for those summary statistics. It may be that the representation mechanism for the summary statistics comprises multiple cognitive processes, and there could be some common underlying processes across perceptual domains.

In addition to the primary purpose, we tested whether representing the mean and variance of complex items could be performed with equivalent precision as observed for simple items such as discs and Gabor patches. We introduced familiar and realistic items, namely illustrations of strawberries for the size domain and those of lollypops for the orientation domain. Our results demonstrated that observers could discriminate the mean and variance between sets of illustrations with precision equivalent to that for simple items. Thus, the findings provide evidence that the *statistical summary representation* could extend to more realistic and natural objects than discs and Gabor patches. Objects in the natural world are multidimensional, and they differ in colour, size, shape and other factors. When we consider that the *statistical summary representation* ability evolved and developed in such an environment, it is to be expected that the *statistical summary representation* is effective with more natural, complex items than discs and Gabor patches. Our findings are noteworthy because few studies have attempted to prove this point. It should be noted that the sizes of the components of the strawberry illustrations are correlated. When the overall size of the strawberry was increased, the red, green and black component areas were increased by the same ratio. This may help observers to derive the mean value.

Moreover, we tested the relationship between performances in the mean and variance tasks in the size and orientation domains. We predicted that if different perceptual domains share a common mean representation system, then an individual who could precisely represent the mean size of an item set should also be able to precisely represent the mean orientation of the set. In other words, there would be considerable correlation between performances in the mean size and orientation tasks. Our result revealed a significant correlation between the individual performances in the size and orientation mean tasks, suggesting that a common representation system exists across perceptual domains. In relation to this issue, Haberman et al. (2015) found no correlation between low- and high-level perceptual domains (e.g. orientation and facial expression), whereas they found correlation within the respective low- (e.g. orientation and colour) and high-level perceptual domains (e.g. person identity and facial expression). Thus, they claimed that the *statistical summary representation* is not derived by a single, domain-general process. The relationship between perceptual domains depends on how proximal they are in the space. In line with their argument, our results suggest that orientation and size domains are close to each other in perceptual space and that the statistical summary mechanism for each domain are close or similar enough to result in similar output results, as orientation and size are considered low- and middle-level perceptual domains, respectively. We also tested whether estimation of the variance of items in a set shares a general representation mechanism across perceptual variables. We found a mild correlation between performances in the size and the orientation variance tasks, although nonsignificant. There may be a common process for variance representation across perceptual domains. As only a few studies have tested variance, further investigations are necessary to draw definitive conclusions on this matter.

Additionally, we obtained the observers’ introspections while they performed each task. The results demonstrated that the observers used various strategies to extract the mean and variance, even with a short observation time of 240 ms, and that there were large individual differences in strategies across tasks and perceptual domains. The findings imply that there might be some conscious and focused attention process in the *statistical summary representation*, although automatic and distributed attention processes in the *statistical summary representations* were claimed in previous studies (Chong & Treisman, 2003, 2005). It should be noted that the introspections of observers might not necessarily reveal the representation processes. Nevertheless, they revealed how observers performed each task, at least at the level of consciousness.

It should be noted that we used the undergraduates and postgraduate students as the observers, it may be that the variance in the sample may be too low to find correlations. It is necessary to use a larger variety of observers in future studies.

Further investigations are necessary to support the findings of this study more explicitly. The following three investigations should be considered. First, it will be effective to test whether the practice effect in the mean task transfers to performance in the variance task. If the representation of variance is independent of the representation of the mean, the practice effect in the mean task would not transfer to the performance of the variance task. Simultaneously, if the representation variance involves a common process across feature domains, the practice effect in one task would be transferred to the second task. Second, a post-cue method in which observers are informed of the demands of the task after viewing stimuli in each trial should be tested. In the post-cue procedure, the observers do not prepare a particular strategy for the task (i.e. mean or variance task) prior to stimulus presentation. If the mean and variance share a common representation mechanism, then the performance in the post-cue procedure may not differ from that in the pre-cue procedure. Third, to generalise the present results, it is necessary to use different types of visual properties such as colour, position and speed. As we mentioned in the Introduction section, the present study concerns types of stimuli used in the cognitive approach. It is, however, necessary to use stimuli set such as larger number of random dots, of moving dots and textures, which are used in the psychophysical approach.

In summary, our results revealed three important characteristics in representing summary statistics. First, representations of the mean and variance may involve distinct processes, at least for size and orientation domains. Second, size and orientation may share a common mechanism for representing the mean and possibly for representing variance. Third, despite individual differences, human observers can extract the mean and variance of complex items featuring multiple dimensions. To provide a clear description of the *statistical summary representation(s)*, further work is required to determine the relationship between different summary statistics across various perceptual domains.
